# The type III neurofilament peripherin is expressed in the tuberomammillary neurons of the mouse

**DOI:** 10.1186/1471-2202-9-26

**Published:** 2008-02-24

**Authors:** Krister S Eriksson, Shengwen Zhang, Ling Lin, Roxanne C Larivière, Jean-Pierre Julien, Emmanuel Mignot

**Affiliations:** 1 Psychiatry and Behavioural Sciences, Stanford University, Palo Alto, CA, USA; 2 Research Centre of Centre Hospitalier Universitaire de Québec, Department of Anatomy and Physiology of Laval University, Québec, Canada; 3 Howard Hughes Medical Institute, Stanford, CA, USA; 4 Center for Narcolepsy, Stanford University, 701B Welch Road, Room 145, Palo Alto, CA 94304-5742, USA

## Abstract

**Background:**

Peripherin, a type III neuronal intermediate filament, is widely expressed in neurons of the peripheral nervous system and in selected central nervous system hindbrain areas with projections towards peripheral structures, such as cranial nerves and spinal cord neurons. Peripherin appears to play a role in neurite elongation during development and axonal regeneration, but its exact function is not known. We noticed high peripherin expression in the posterior hypothalamus of mice, and decided to investigate further the exact location of expression and function of peripherin in the mouse posterior hypothalamus.

**Results:**

*In situ *hybridization indicated expression of peripherin in neurons with a distribution reminiscent of the histaminergic neurons, with little signal in any other part of the forebrain. Immunocytochemical staining for histidine decarboxylase and peripherin revealed extensive colocalization, showing that peripherin is produced by histaminergic neurons in all parts of the tuberomammillary nucleus. We next used histamine immunostaining in peripherin knockout, overexpressing and wild type mice to study if altered peripherin expression affects these neurons, but could not detect any visible difference in the appearance of these neurons or their axons.

Peripherin knockout mice and heterozygotic littermates were used for measurement of locomotor activity, feeding, drinking, and energy expenditure. Both genotypes displayed diurnal rhythms with all the parameters higher during the dark period. The respiratory quotient, an indicator of the type of substrate being utilized, also exhibited a significant diurnal rhythm in both genotypes. The diurnal patterns and the average values of all the recorded parameters for 24 h, daytime and night time were not significantly different between the genotypes, however.

**Conclusion:**

In conclusion, we have shown that peripherin is expressed in the tuberomammillary neurons of the mouse hypothalamus. Monitoring of locomotor activity, feeding, drinking, and energy expenditure in mice either lacking or overexpressing peripherin did not reveal any difference, so the significance of peripherin in these neurons remains to be determined. The complete overlap between histidine decarboxylase and peripherin, both the protein and its mRNA, renders peripherin a useful new marker for histaminergic neurons in the hypothalamus.

## Background

Peripherin, a type III neuronal intermediate filament protein, is widely expressed in neurons of the peripheral nervous system (PNS). It is also expressed in a restricted set of brain stem and spinal cord neurons that usually project towards peripheral structures. Most notably, expression of peripherin was demonstrated in several motor nuclei of cranial nerves, ventral horn motor neurons and in the cholinergic laterodorsal tegmentum (LDT) and pedunculopontine tegmentum (PPT) nuclei. In addition, a few unidentified neurons of the posterior hypothalamus contained peripherin, whereas it was absent from the major monoaminergic nuclei of the brainstem and midbrain [1-3].

Peripherin can self-assemble or co-assemble with other neurofilament subunits to form intermediate filament networks [4]. Developmental expression peaks during the axonal growth phase and decreases postnatally [5], suggesting a role in neurite elongation during development. The expression of peripherin is also upregulated after neuronal injury e.g. in motor neurons and dorsal root ganglia after peripheral axotomy, supporting a role for this protein in axonal regrowth [6,7]. It should be noted, however, that in spite of the fact a majority of spinal cord motoneurons express peripherin, peripherin knockout (KO) mice exhibit no overt phenotype, develop, survive and reproduce normally. Motoneuron morphology, neurite outgrowth and axonal path finding also appear normal in these mice, although a subset of small unmyelinated sensory fibers are lost. This may be explained by redundancy and the presence of type IV intermediate neurofilaments in many neurons. The exact function of peripherin is thus not known [8].

Overexpression of peripherin interferes with anterograde axonal transport. As a result, neurofilament aggregates form in neuronal somata and axons, ultimately producing spinal motor neuron death in overexpressing transgenic mice [9,10]. Such an accumulation of peripherin-containing neurofilaments has also been demonstrated in degenerating spinal motor neurons of amyotrophic lateral sclerosis patients and in the murine model of this disease, i.e., the transgenic mice expressing a mutant of the superoxide dismutase-1 (SOD1G37R), a motor neuron disease characterized by loss of motor neurons resulting in paralysis and death [11-13].

Peripherin may also be involved in the pathology of insulin-dependent diabetes mellitus (IDDM). Indeed, the protein is a known target autoantigen in the nonobese diabetic mouse model, where islet cell infiltrating B-cell clones reactive to peripherin have been identified early in the course of the disease. Peripherin is produced by both the PNS and, in young animals, by islet β cells, so it is conceivable that this immune response is involved in the destruction of both PNS elements and islet β cells in IDDM, although no direct involvement of this antigen has yet been shown in human patients [14].

During a microarray experiment comparing the gene expression of the posterior hypothalamus with that of the lateral hypothalamus in mice, we found a sixty-fold higher expression of peripherin in the posterior hypothalamus. We therefore have used *in situ *hybridization (ISH), immunocytochemistry (ICC) and *in vivo *recordings in mice overexpressing peripherin [9], peripherin KO mice [8] and their littermates to study the expression and function of peripherin in the posterior hypothalamus. We report that peripherin is expressed by the histaminergic tuberomammillary (TM) neurons of the posterior hypothalamus and that KO and transgenic animals exhibit no obvious phenotype.

## Results

### Neuroanatomical studies

**Figure 1:**
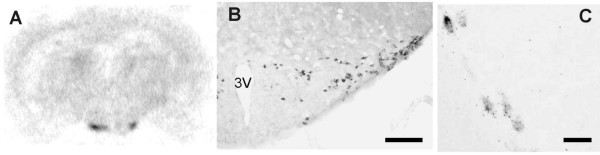
***In situ *hybridization of peripherin**. The expression of peripherin mRNA was studied with ISH. The autoradiogram in A shows a mouse brain section hybridized with a ^35^S-labelled peripherin probe. The selective staining of the TM nucleus is evident. In B, a digoxigenin labelled mRNA probe reveals the distribution of peripherin expressing neurons in detail. The distribution is very similar to the histaminergic TM neurons. In C, neurons of the lateral TM that were hybridized simultaneously with a digoxigenin labelled probe for peripherin and a ^35^S-labelled probe for HDC are shown. The alkaline phosphatase staining for peripherin, which differentiate the neurons from the background, is colocalized with the silver grains that indicate the localization of HDC expression. 3 V, third ventricle. Scale bar 100 μm in B, 20 μm in C.

ISH with a ^35^S-labelled probe indicated expression of peripherin in the posterior hypothalamus (Fig 1A). Staining with a digoxigenin labelled probe demonstrated a distribution of the peripherin signal in neurons with a distribution reminiscent of the histaminergic neurons (Fig 1B). Double ISH staining with a digoxigenin labelled peripherin probe and a ^35^S-labelled probe for the histamine-synthesizing enzyme histidine decarboxylase (HDC) indicated colocalization, showing that peripherin is expressed in the histaminergic TM neurons (Fig 1C). We did not detect reliable expression of peripherin in any other part of the forebrain. Peripherin mRNA signal was absent in peripherin KO mice.

**Figure 2:**
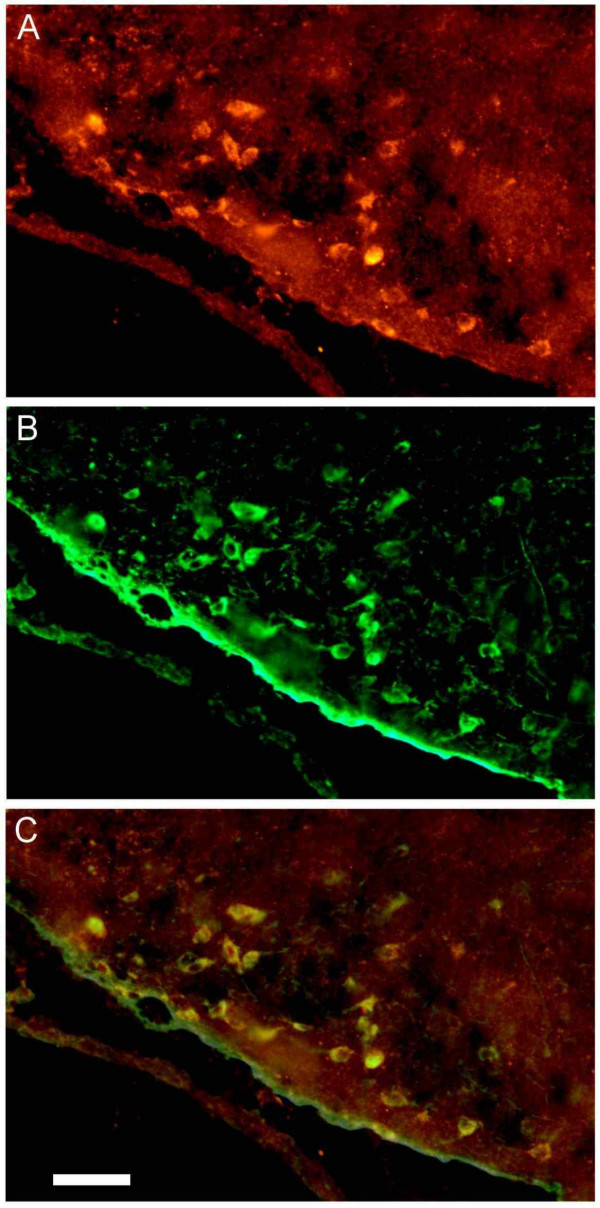
**Immunostaining of HDC and peripherin**. We preformed double immunocytochemistry for HDC and peripherin. There is an extensive colocalization of the two proteins in the TM neurons. In A, the ventral/lateral part of the TM nucleus is stained for HDC in red. In B the same area is stained for peripherin (green). The staining for the two proteins is digitally merged in C. Neurons that are stained for both HDC and peripherin appear yellow in this picture. Most neurons are stained for both antigens. Scale bar 20 μm.

Double ICC staining for HDC and peripherin revealed extensive colocalization, showing that peripherin immunoreactivity is located in histamine-producing neurons across the entire TM nucleus (Fig 2). Almost all (> 95%) TM neurons were stained for both proteins, although single neurons stained for only one of the proteins were occasionally seen. This lack of staining was usually seen when the general staining for the antigen was weak and may thus have been caused by low sensitivity. Neurons that stained only for peripherin were only occasionally seen in this part of the brain.

Histamine ICC was used to study whether altered peripherin expression would affect TM neurons and their processes. After histamine ICC, comparisons between peripherin KO, overexpressing and WT mice did not reveal any visible difference in the appearance of TM neurons or in the density of histamine immunoreactive axons in any of the studied brain regions.

### Measurement of locomotor activity, feeding, drinking, and energy expenditure in peripherin KO animals

Central histamine has been implicated in the regulation of sleep-wake regulation, cognition, metabolism, and appetite regulation. Three months old female and male heterozygotic (Hz) and homozygotic (H) littermates were introduced to the recording chambers at Zeitgeber time (ZT) 10 (*i.e*., two hours before lights-off). There was no significant difference in body weight between the Hz and H mice (Table 1). Upon entering the chambers, all mice displayed a brief period of exploratory behaviour and the level of locomotor activity (LMA) gradually returned to daytime baseline after 1 hour. This adaptation period was similar between both genotypes (data not shown). Both genotypes displayed diurnal rhythms in LMA, drinking, feeding and energy expenditure with all the parameters higher during the dark period and lower during the light period. The respiratory quotient (RQ), an indicator of the type of substrate being utilized, also exhibited a significant diurnal rhythm in both genotypes. During the day, when mice are mostly resting and not feeding, the RQ was lower, indicating a higher fat utilization. The diurnal patterns and the average values of all the parameters for 24 h, daytime and nighttime were not significantly different between the genotypes (Table 1). Two-way ANOVA revealed significant differences in LMA, drinking and energy expenditure (EE) between sexes, whereas no interaction between sex and genotype was found.

**Table 1:** Parameters of peripherin KO mice measured in metabolic chambers (Mean ± SEM)

	Female H (n = 6)	Female Hz (n = 6)	Male H (n = 5)	Male Hz (n = 5)
Body weight (g)				
	24.1 ± 0.8	23.7 ± 1.1	32 ± 1	30.5 ± 0.5
				
LMA (count/h)				
Day	1922 ± 161	1734 ± 174	1289 ± 182	1135 ± 129
Night	5102 ± 725	4764 ± 476	3010 ± 536	2278 ± 196
24 h	3512 ± 415	3249 ± 307	2150 ± 353	1707 ± 150
				
Food intake (g)				
Day	1.6 ± 0.1	1.1 ± 0.2	1.2 ± 0.2	1.2 ± 0.1
Night	3.1 ± 0.2	3.1 ± 0.4	2.7 ± 0.2	2.6 ± 0.3
24 h	4.7 ± 0.3	4.2 ± 0.4	3.8 ± 0.3	3.8 ± 0.3
				
Water intake (mL)				
Day	1.4 ± 0.1	1.6 ± 0.2	1.0 ± 0.1	0.7 ± 0.2
Night	3.8 ± 0.6	4.3 ± 0.6	2.2 ± 0.2	2.1 ± 0.3
24 h	5.2 ± 0.7	5.9 ± 0.8	3.3 ± 0.3	2.8 ± 0.4
				
EE (kcal/h)				
Day	0.75 ± 0.05	0.72 ± 0.02	0.53 ± 0.01	0.52 ± 0.03
Night	0.89 ± 0.05	0.87 ± 0.03	0.58 ± 0.02	0.58 ± 0.02
24 h	0.82 ± 0.05	0.79 ± 0.02	0.56 ± 0.01	0.55 ± 0.02

**Figure 3:**
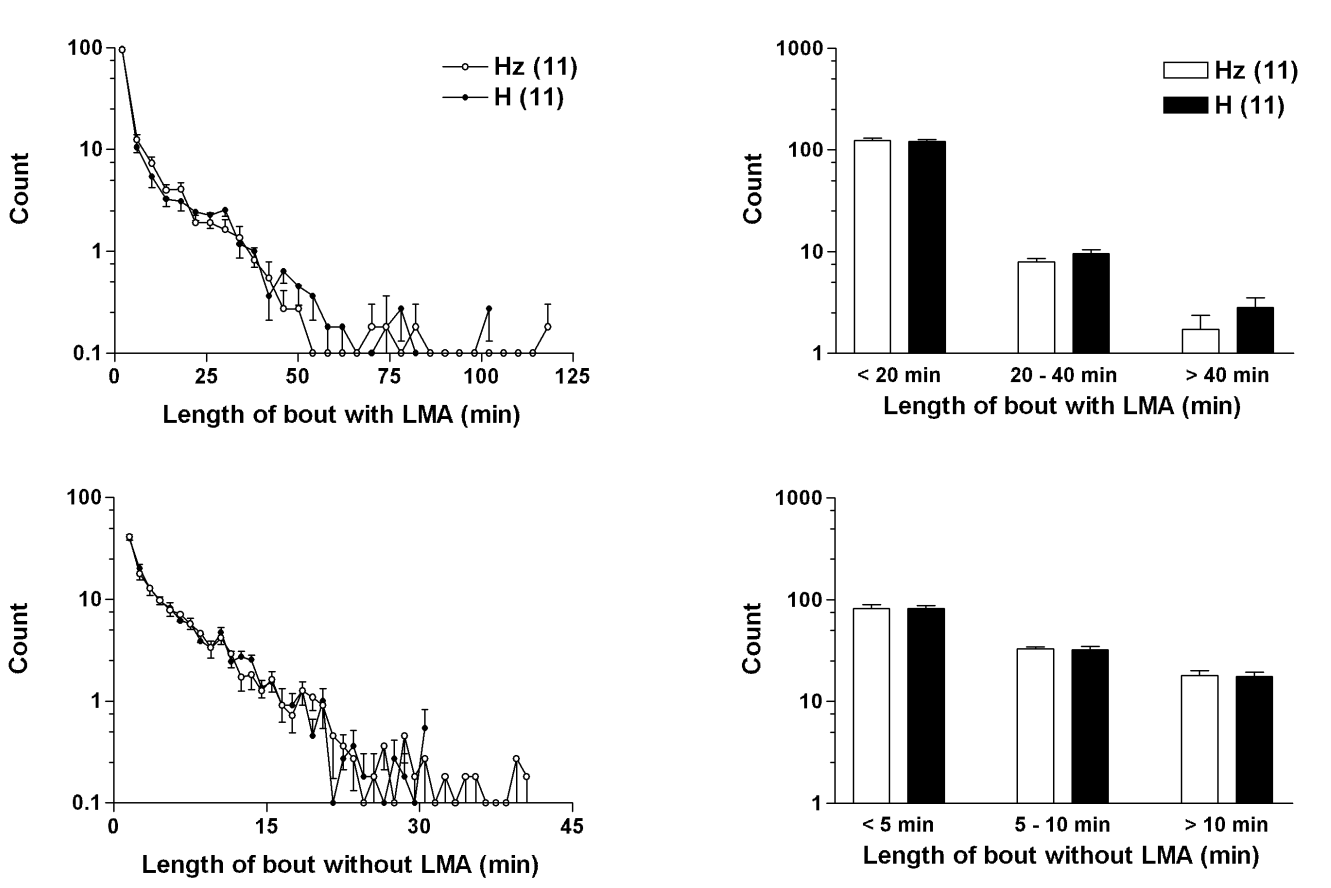
**Measurement of locomotor activity**. Histograms of active (top) and quiet (bottom) bout duration in peripherin heterozygotic (Hz) and homozygotic (H) mice. Locomotor activity data were collected at 1 minute intervals. Each minute with LMA larger than 6 counts was defined as being active, and the rest were defined as being quiet. A bout was defined as a period during which a state presented consecutively without interruption of the other. For equal bin histograms (left), the active bout data are binned in 4-minute intervals, and the quiet bout data are binned in 1-minute intervals. The solid lines with white circles represent group averages of the Hz mice and the solid lines with black circles represent group averages of the H mice. The bar graphs (right) show the differences between the Hz and H mice in three arbitrary bins of bout duration. The white bars represent the Hz mice and the black bars represent the H mice. A similar analysis of LMA in 8 hypocretin-ataxin mice versus 8 control littermates was effective in predicting sleep/wake fragmentation hypocretin-ataxin mice (see text).

As the other physiological parameters didn't suggest any major behavioral differences, and due to a lack of sufficient numbers of animals, sleep was not directly accessed in these animals. As a proxy, we measured LMA at 1 min interval and studied the distribution of active (wake) and inactive (sleep and quiet wake) bouts during the day and night [15]. This algorithm was tested in hypocretin-ataxin mice that display significant sleep/wake fragmentation compared to wild type (WT) littermates. As these mice were included solely to validate the algorithm, we decided that it was sufficient to only test it on female mice. As expected the active/inactive state distribution was significantly shifted to shorter active and inactive periods. According to this algorithm, each minute with an activity level of more than 6 counts is considered as active state, and the rest is quiet states. The duration and number of each continuous state is then calculated, a distribution plotted (Fig. 3) and subjected to statistical analysis. In comparing narcoleptic and control mice, we found that episodes of short duration (< 20 min) were significantly more frequent in ataxin-hypocretin transgenic (TG) than in WT mice (150.3 ± 6.3 vs. 118.6 ± 11.9, p = 0.035, n = 8 each) while active episodes of long, consolidated, duration (> 40 min) were less frequent in TG than in WT mice (1.8 ± 0.6 vs. 4.5 ± 0.7, p = 0.011.05, n = 8 each). Conversely, inactive episodes of short duration (< 5 min) were significantly less frequent than in WT mice (124.3 ± 6.4 vs. 88.3 ± 12.8, p = 0.025, n = 8 each). These results validated the use of activity bout measurements as a partial proxy for the study of sleep, as these results closely mimics sleep/wake distribution abnormalities in these mice based on traditional sleep recording [15]. In contrast to the results obtained above, when we compared histograms and means for the peripherin KO Hz and H mice, we could not detect any significant difference in LMA or any of the other recorded parameters (Fig. 3).

## Discussion

In this study we have shown that histaminergic TM neurons produce peripherin, a neurofilament mainly expressed in neurons with long axonal projections to the periphery. TM neurons send out axons that innervate most parts of the brain and, at a lower density, the spinal cord [16,17]; innervation of peripheral targets by these neurons has never been described. Expression of peripherin in TM neurons suggested that these neurons may need this protein for elongation or projection path-finding. To test this hypothesis, we used immunostaining to study histaminergic axons in the brain of peripherin KO and over-expressing mice, but we could not observe any difference in the distal innervation in any of the studied regions. This negative result is in agreement with the findings of Larivière and co-workers [8], who studied the KO mice and, apart from the absence of a subset of spinal sensory axons, found no morphological abnormalities in these animals.

TM neurons have been implicated in the regulation of waking and feeding behaviours [18], and their activity is strongly associated with behavioural state; they fire tonically in a regular pattern during waking but not at all during deep slow wave sleep and rapid eye-movement sleep [19]. Inhibition of histamine release or treatment with histamine H_1 _receptor antagonists lead to an increase in cortical slow activity and increased sleep, whereas increased histamine signalling induces cortical desynchronization and decreases sleep [20-23]. Furthermore, mice lacking either HDC or the H_1 _receptor are incapable of prolonged activity and wakefulness, most notably in a novel environment, and also have a disturbed circadian rhythm of locomotor activity [24,25]. There is also evidence that histamine is involved in the control of feeding. Indeed, increased CNS histamine concentration decreases feeding in rats, whereas feeding is increased by H_1 _receptor antagonists or by inhibition of histamine synthesis [26,27].

Larivière and co-workers [8] did not observe any obvious phenotype in peripherin KO mice. They did not measure activity level, however, so to further explore the possibility of a behavioural phenotype in these mice, we studied the circadian pattern in LMA, energy expenditure, feeding and drinking. We could not detect any significant difference between peripherin KO mice and controls, however, so the function of peripherin in the TM and other neurons that could influence such behaviors will need more study. Earlier studies suggest a role for peripherin in axonal elongation and repair, and there is also some evidence for a role in hippocampal synaptic plasticity [28], and transient events, such as axonal rewiring or synaptic reorganization, which also could be expected to cause rather subtle changes, could have gone undetected in our study. It is also possible that we would have found a difference between KO and control mice if we had used some type of behavioral or pharmacological challenge or sleep deprivation.

It was recently demonstrated that many islet-infiltrating B-cells in IDDM produce antibodies that are specific for peripherin and therefore recognize the PNS, which could explain the neuropathy that is commonly seen in IDDM [14]. Another protein that is produced by islet β-cells as well as many CNS neurons, glutamic acid decarboxylase (GAD), is an important autoantigen in both IDDM and stiff-man syndrome, an uncommon CNS disorder caused by impaired GABAergic neurotransmission that often coexist with IDDM [29,30]. It is not known whether peripherin autoimmunity may cause damage to the PNS or CNS, but if the autoimmune mechanisms directed towards peripherin leads to attacks on CNS neurons in the same manner as GAD-specific antibodies target GABAergic neurons, the TM neurons could be especially susceptible, as they in addition to peripherin also express GAD [31].

The expression of peripherin in the brain stem and spinal cord is largely restricted to cholinergic neurons. Thus, peripherin immunoreactive neurons include autonomic motor neurons, preganglionic sympathetic neurons, neurons in the PPT and LDT, as well as brain stem and spinal cord motor nuclei [1]. This, together with the recent finding that the TM neurons express a peripheral splice variant choline acetyl transferase, [32] hint at the possibility that the expression of peripherin is somehow linked to cholinergic neurotransmission.

For the double ICC stainings we needed a marker that stains all TM neurons selectively. There are several markers for these neurons available. Antiserum against histamine stains these neurons very well [16], but the best antiserum available needs a special fixative (see methods), which is incompatible with some antigens and, as we found out, this includes peripherin. The TM neurons also contain GAD and GABA. Antisera against these two compounds tend to be difficult to use and are not selective for the TM neurons, as there are other GABAergic neurons close to the TM nucleus. Antisera against adenosine deaminase stain TM neurons in the rat [33], but to our knowledge this has not been reported from any other species, and in our experience these antisera don't stain mouse TM neurons at all. There are several neuropeptides present in the TM neurons, but there is considerable interspecies variation and antisera against these peptides tend to only stain a fraction of the TM neurons [31]. In the light of the above, HDC, and possibly GAD or GABA, were the only markers we could think of that would stain all the TM neurons in formalin-fixed mouse tissue. Therefore, we think that peripherin will be a useful addition to the repertoire of markers for TM neurons, as it is highly specific for these neurons in the hypothalamus.

## Conclusion

In conclusion, we have shown that peripherin is expressed in the TM neurons of the mouse hypothalamus. We could not detect any neuroanatomical or behavioural phenotype in mice either lacking or overexpressing peripherin, so the significance of peripherin in TM neurons remains to be determined. The complete overlap between HDC and peripherin, both the protein and its mRNA, makes it a useful new marker for TM neurons.

## Methods

### Mice and tissue preparation

Experiments were performed using peripherin KO mice of mixed 129/C57Bl/6 background that have a targeted disruption of the peripherin gene [8], peripherin overexpressing TG mice of C57Bl/6 genetic background carrying 20 copies of the peripherin gene [9] and their WT littermates. Ordinary C57BL/6J mice were used in some double stainings due to a shortage of littermates. Three female 10–15 weeks-old mice of each genotype were given an overdose of pentobarbital and perfused transcardially with either 10% neutral buffered formalin or, for histamine ICC, wit h 4% 1-ethyl-3(3-dimethyl-aminopropyl)-carbodiimide and 0.2% *N*-hydroxysuccinimide (Sigma-Aldrich, St. Louis, MO) in 0.1 M phosphate buffer, pH 7.4. Brains were removed and immersed in the same fixative over night, cryoprotected, and cryosectioned at 15–25 μm thickness.

Hypocretin-ataxin TG mice (N8 and beyond, thus considered pure C57Bl/6) expressing a truncated Machado-Joseph disease gene product (ataxin-3) with an expanded polyglutamine stretch specifically in hypocretin neurons [34] were used to develop the algorithm for predicting sleep/wake pattern based on LMA data measured. The expression of ataxin-3 in hypocretin-expressing neurons causes cell death, and the transgenic mice display significant sleep/wake fragmentation compared to their wild type littermates [15]. Eight female TG mice of age 7 months and 8 WT female littermates were used for this study.

All efforts were made to minimize animal suffering and to reduce the number of animals used. The study was approved and conducted in accordance with the guidelines of Stanford's Administrative Panel for Laboratory Animal Care.

### *In situ *hybridization

Cloned cDNAs corresponding to the complete coding sequence of the mouse peripherin mRNA (IMAGE clone ID 6475506, [Genbank:BC046291]) and HDC mRNA (IMAGE clone ID 5361831, [Genbank: BI732768] were purchased from Invitrogen; inserts were sequence verified. After purification using the Qiaprep Miniprep Kit (Qiagen), plasmids were linearized with EcoRI and transcribed with T7 polymerase using the Riboprobe System (Promega, Madison, WI) and ^35^S-UTP (Amersham/GE Healthcare Bio-Sciences, Piscataway, NJ) or digoxigenin-UTP (Roche Diagnostics, Indianapolis, IN) to produce antisense riboprobes.

Probes were diluted in hybridization buffer comprising of 600 mM NaCl, 10 mM Tris-HCl (pH 7.5), 1 mM EDTA 10% dextran sulphate, salmon sperm DNA 0.01%, yeast RNA 0.005, 1× Denhardt's solution, 0.01% SDS, 0.01% sodium thiosulphate, 100 mM DTT (all reagents from Sigma) and 50% formamide (Fischer Scientific, Pittsburgh, PA). Radiolabeled riboprobes were diluted to give 3 × 10^4 ^counts/μl, whereas digoxigenin-labelled probes were diluted 1:500. Sections were pretreated for 10 min in citrate buffer (pH 6.0) at ~95°C, and hybridized for 12–16 hr at 54°C. After RNase A (20 μg/ml; Sigma) treatment for 30 min at 37°C, the tissue was washed in 2×SSC at 50°C for 1 hour, in 0.2×SSC at 55°C for 1 hour and in 0.2×SSC at 65°C for 1 hour. After dehydration, slides with ^35^S probes were exposed to X-ray films for 1 to 40 days, whereas slides hybridized with digoxigenin probes were immunostained. For immunostaining, slides were treated with 3% normal sheep serum and 0.1% Triton X-100 in phosphate-buffered saline (pH 7.3; PBS) for 3 hours, followed by overnight incubation in alkaline phosphatase-conjugated sheep anti-digoxigenin antibodies (1:5000 dilution; Roche). After blocking of endogenous alkaline phosphatase in 0.1% levamisole for 5 min, blue-purple reaction products were visualized by incubation in 0.3 mg/ml nitro blue tetrazolium and 0.2 mg/ml 5-bromo-4-chloro-3-indolyl phosphate (Roche) in GB3 buffer (pH 9.5) at RT for 12–36 hours. Some slides were hybridized with both a ^35^S-labelled and a digoxigenin-labelled probe. After alkaline phosphatase staining, these slides were first covered with parlodione and then dipped in liquid NTB2 emulsion (Kodak, Rochester, NY) and exposed in darkness for 3–6 weeks.

### Immunocytochemistry

Antibody incubations and washes were done at room temperature in PBS with 0.25% Triton X-100. Antibody solutions all contained 2% donkey serum. The tissue was treated with 1% H_2_O_2 _for 15 min to quench endogenous peroxidase, blocked with 5% normal donkey serum for 2–4 hours and incubated overnight with primary antiserum. Double staining for HDC and peripherin was done in sequence. The first staining was done using guinea pig-anti-HDC serum (1:200 overnight; American Research Products, Belmont, MA), biotinylated donkey-anti-guinea pig (1:500 for 90 min; Jackson Immunoresearch, West Grove, PA), avidin-biotin complex (1:500 for 90 min; Vector Laboratories, Burlingame, CA), biotinylated tyramide (1:50 for 10 min; Perkin Elmer, Boston, MA) and Alexa Flour 555-conjugated streptavidin (1:500 for 90 min; Molecular Probes, Eugene, OR). After HDC staining, tissue was treated with 1 H_2_O_2 _for 45 min and the avidin-biotin blocking kit (Vector) to prevent interference with the second staining. The tissue was then incubated with rabbit-anti-peripherin (1:3000 overnight; Chemicon, Temecula, CA), biotinylated donkey-anti-rabbit (1:500 for 90 min; Jackson), avidin-biotin complex (1:500 for 90 min), biotinylated tyramide (1:50 for 10 min) and Alexa Fluor 488-conjugated streptavidin (1:200 for 90 min; Molecular Probes). Single stainings of each protein were done to verify results. Stainings where either primary antiserum were excluded were also done to examine the possibility of cross-reactions. For histamine a rabbit-anti-histamine serum (1:500; Chemicon) was used. After washing, slides were incubated with Alexa Fluor 488-conjugated donkey-anti-rabbit serum (Molecular Probes). All steps were performed at RT and slides were washed for at least 20 min after each step. Finally, micrographs of the alkaline phosphate staining for mRNA and fluorescence stainings were taken with a microscope equipped with a digital camera.

### Measurement of locomotor activity, feeding, drinking, and energy expenditure

Mice (for peripherin KO line, 6 Hz females, 6 H females, 5 Hz males, 5 H males, age 3–4 months; for hypocretin-ataxin transgenic line, 8 WT females, 8 TG females, age 7 month old) were introduced to an 11 × 31 × 12 cm Plexiglas recording chamber for indirect calorimetric measurement of oxygen consumption and carbon dioxide production (Oxymax, Columbus Instruments, Columbus, OH). Room air was supplied to the chamber at a flow rate of 570 ml/min, and was referenced every hour during measurement. Each chamber was equipped with real-time monitoring of powdered food intake, cyclic measurements of water intake through volumetric detectors and of LMA through infrared monitors. This system allows simultaneous measurement of 16 animals at a time with indirect calorimetric measurements cycling through the chambers once every hour, measuring one chamber at a time. Mice spent at least 48 hours adapting to the environment; data collected after this adaptation period were used in the analyses. Bodyweight was measured before and after each experiment to ensure animal health.

Feeding, drinking, and energy expenditure data were all adjusted for a body weight of 30 g. Energy expenditure was calculated from measured oxygen consumption (VO_2_) and carbon dioxide production (VCO_2_): EE = 3.815 × VO_2 _+ 1.232 × VCO_2_. Respiratory quotient was defined as: RQ = VCO_2_/VO_2_. Data were analyzed with Student's t-test and two-way ANOVA.

## List of abbreviations

EE, energy expenditure; GAD, glutamic acid decarboxylase; H, homozygotic; HDC, histidine decarboxylase; Hz, heterozygotic; ICC, immunocytochemistry; IDDM, insulin-dependent diabetes mellitus; ISH, *in situ *hybridization; KO, knockout; LDT, laterodorsal tegmentum; LMA, locomotor activity; PNS, peripheral nervous system; PPT, pedunculopontine tegmentum; RQ, respiratory quotient; TG, transgenic; TM, tuberomammillary; WT, wild type; ZT, Zeitgeber time

## Authors' contributions

KSE did the *in situ *hybridization, immunostaining and drafted the manuscript, SZ did the activity recordings and drafted parts of the manuscript, LL helped with the tissue preparation and genotyping, RCL and JPJ provided the transgenic animals and EM conceived of the study and participated in the design, coordination and manuscript writing. All authors read and approved of the final version of the manuscript.
